# Maggot debridement therapy for burns surgery avoidance in an elderly and comorbid patient: A case report

**DOI:** 10.1177/20595131241279076

**Published:** 2025-03-21

**Authors:** Alyss Vaughan Robinson, Hester Lacey, Baljit Dheansa

**Affiliations:** * These authors contributed equally as co-first authors.; 1 8962 Queen Victoria Hospital NHS Foundation Trust, East Grinstead, UK; 2 12190 Brighton and Sussex Medical School, Brighton, UK; 3 University Hospitals Sussex NHS Foundation Trust, Brighton and Hove, UK

**Keywords:** Larvae, maggot debridement therapy, eschar, burns, biodebridement, full thickness

## Abstract

**Introduction:**

Maggot debridement therapy is an effective and widely used biodebridement method in chronic or non-healing wounds but is infrequently documented in burn injuries. Many burn patients wish to avoid surgical intervention, and in an ageing population with increasing comorbidities surgery may not always be preferable. Here we describe its successful use in an elderly and comorbid patient.

**Methods:**

The larvae were applied to a 0.5% full thickness burn wound on the thigh using two treatments of BioMonde Biobags, and he achieved healing within eight weeks.

**Discussion:**

Maggot debridement therapy has been documented to shorten healing time, increase the likelihood of healing, and reduce antibiotics use in other chronic wounds. Maggots may be more selective in debriding wounds than sharp surgical debridement, preserving more healthy tissue. There is evidence to suggest that maggots clear biofilms created by *Staphylococcus aureus* and *Pseudomonas aeruginosa*, which are common organisms cultured in burn wounds. The patient was enthusiastic about the therapy and would recommend it to other patients.

**Conclusions:**

More formal evidence is required to compare outcomes between maggot debridement therapy and surgical intervention in such patient subgroups, as this may become a workhorse therapy for successful burns debridement and treatment.

**Lay Summary:**

Burn injuries are common and increasingly so in the elderly. Full-thickness injuries are those which involve all the layers of skin and are at risk of becoming long-term wounds if left to heal on their own. These types of wounds will often develop a hard covering layer, called eschar, which protects the regenerating skin underneath but can slow down how fast the wound heals. Often patients with full thickness injuries will need the eschar removed, the wound surgically cleaned (known as debriding) and a skin graft to reduce the healing time. However, in elderly patients with medical issues such as diabetes and heart problems (as in this case), surgery may not be advisable due to the risks of having anaesthetics, as well as the medical problems possibly impacting on how well the skin graft will work. Maggots are immature green-bottle fly larvae which feed on dead tissue and release enzymes to break it down to digest. They have been used in wound care for centuries but are less frequently considered an option for burns. In this case report, an elderly and comorbid patient sustained a deep burn injury to his thigh. He declined surgery and maggots were used instead, which were highly safe and effective. He did not require skin grafting. We suggest more studies are required to compare how effective this treatment is within the elderly population as means of avoiding surgery.

## Introduction

Burn injuries are the fourth most common type of trauma. In the United Kingdom, an estimated 250,000 people sustain burn injuries every year, but only 16,000 require hospital admission.^1^ This is because the vast majority (90%) of burns are described as ‘non-complex’ and can often be managed in an outpatient setting.^2^

The number of burn injuries in the elderly is increasing in line with the aging population of the United Kingdom.^3^ Elderly patients are more likely to develop full-thickness burns, relating to atrophic skin, lack of first aid and delayed presentation to burns services; consequently, a greater proportion of elderly patients require surgical treatment.^3^ Concurrently, elderly patients are more likely to have significant co-morbidities and polypharmacy which may preclude surgical intervention.

Burn wound progression can result in eschar formation, especially in patients who have multiple co-morbidities or in areas of poor perfusion.^4,5^ Eschar needs to be removed before the wound can be successfully closed.^4^ The common approach of surgical debridement may not be possible in those patients who may not be fit for operative treatment.^3^ In such cases, wounds may be temporarily left to undergo autolysis and then granulation is encouraged before definitive wound closure by skin grafting or spontaneous epithelialisation.^6^

Once an eschar develops it can provide temporary wound coverage and protection but can also culture bacteria causing a localised inflammatory response which can result in local or even systemic infection.^4^ Therefore, debridement of eschar is a crucial component of burn wound healing, of which surgery can be effective but may require a theatre visit. However, many patients wish to avoid any surgical intervention and patients with significant comorbidities, such as the elderly, may pose an anaesthetic and bleeding risk and have poorer healing and graft uptake.^3^

Maggot debridement therapy (MDT) is a safe and effective option for debridement of devitalised tissue from necrotic and chronic wounds.^7–11^ It involves the application of *Lucilia sericata* larvae within a biological bag which selectively remove dead tissue and bacteria. MDT is a considered option for traumatic and atraumatic wounds refractory to conservative measures, where surgical management is not possible.^7–11^ There is tangible evidence to support the role of MDT in both effective debridement and disinfectant of necrotic and infected wounds and stimulating and promoting earlier wound healing.^11^ The use of MDT in the debridement of burn eschar has been reported.^12–16^ MDT has been used successfully for debridement of both acute and chronic full thickness burns as a pre-grafting measure, to reduce anaesthetic risk and operative time.^12–16^ The role of MDT for debridement of full thickness burns with extensive, thick eschar as an alternative to surgical management is not well described. We describe a case of MDT used successfully to debride a deep dermal and superficial partial thickness scald to the thigh, scrotum, and groin, complicated by infection and full thickness eschar, in an elderly patient with significant comorbidities keen to avoid surgical management.

This report has been written in accordance with CARE Guidelines 2013 (https://www.care-statement.org/checklist).

## Patient information

A 76-year-old man was referred to our Burns Unit two days after sustaining a scald burn to his left thigh, groin, and scrotum from hot tea. He had not undertaken any first aid measures prior to presentation and was outside of the effective window for cooling.

His past medical history included Type 2 diabetes mellitus, atrial fibrillation, ischaemic heart disease with previous mitral and atrial valve replacement, hypertension, chronic kidney disease and heart failure. Of note his regular medications included insulin and warfarin.

He was very keen to avoid surgery as much as possible due to his medical issues, and willing to accept the risk of prolonged wound healing. Patient consented to use of all images and details for production.

## Results

The burn was assessed as superficial partial thickness to the scrotum and groin and a deep dermal to full thickness burn to the left thigh. The total body surface area (TBSA) was estimated at 1% (see Table 1 for timeline and photographs). The patient elected to take a conservative approach rather than consider early surgery.

**Table 1.** Timeline of events.

	Events	Photograph / Interventions
Day 0	Scald burn to left thigh, groin and scrotum from hot tea at home	No first aid
Day 2	Presented to local minor injuries unit and burns clinic	Dressed with Jelonet, gauze, hypofix (Figure 1)
Day 5	Second presentation to burns clinic Wound appeared cellulitic	Prescribed oral flucloxacillin Dressed with inadine, gauze, hypofix
Day 7	Third presentation to burns clinic Cellulitis improving	Dressed with inadine, gauze, hypofix Wife to continue dressing after showers
Day 14	Fourth presentation to burns clinic 0.5% full thickness area to left thigh with eschar Patient declined surgery	Medihoney apinate, medihoney tulle, gauze hypofix, Dressing at home after showering (Figure 2)
Day 21	Fifth presentation to burns clinic Ongoing thick eschar, decision taken to try larvae	Dressed with hydrogel and duoderm while awaiting arrival of larvae therapy (Figure 3)
Day 30	BioMonde BioBag Larvae applied	Duoderm to edges, sudocreme, larvae, wet gauze, dry gauze, k-lite bandage Daily gauze changes to keep moist
Day 33		Jelonet, gauze, crepe (Figure 4)
Day 34	New BioMonde BioBag Larvae applied	Duoderm to edges, sudocreme, larvae, wet gauze, dry gauze, crepe
Day 37	Larvae removed	Larvae removed, water + soap, medihoney tulle, gauze, bandage, tubifast to secure. Patient to change dressings at home every three days (Figure 5)
Day 51	Clinical review	Medihoney tulle, blue gauze, crepe bandage, hypofix. Change dressings at home every two to three days (Figure 6)
Day 86	Wound healed	Simple non-woven dressing for comfort only (Figure 7)

The copyright for photographs imbedded within this table belong to Queen Victoria Hospital NHS Foundation Trust. These must not be reproduced without seeking consent from qvh.communications@nhs.net.

**Figure 1.**
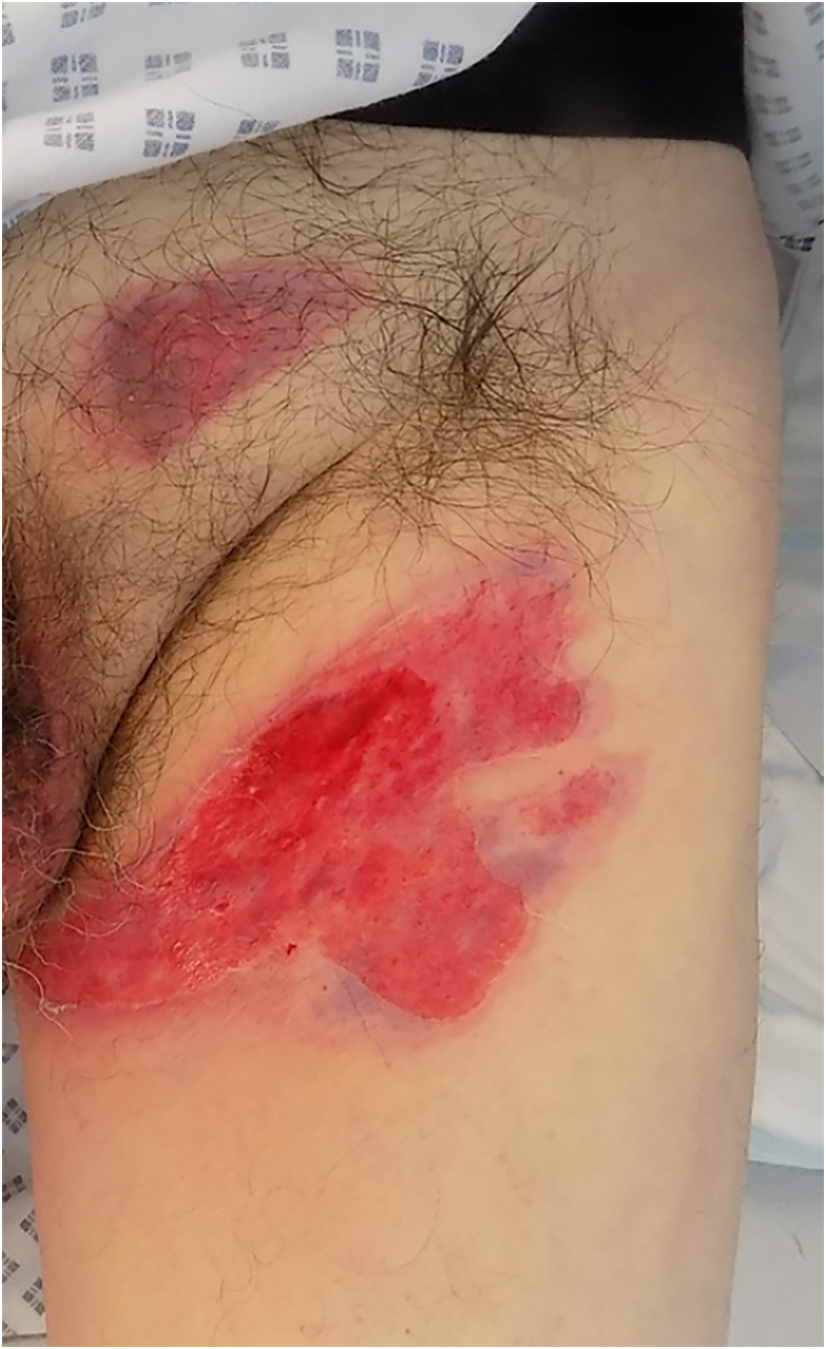
Day 2.

**Figure 2.**
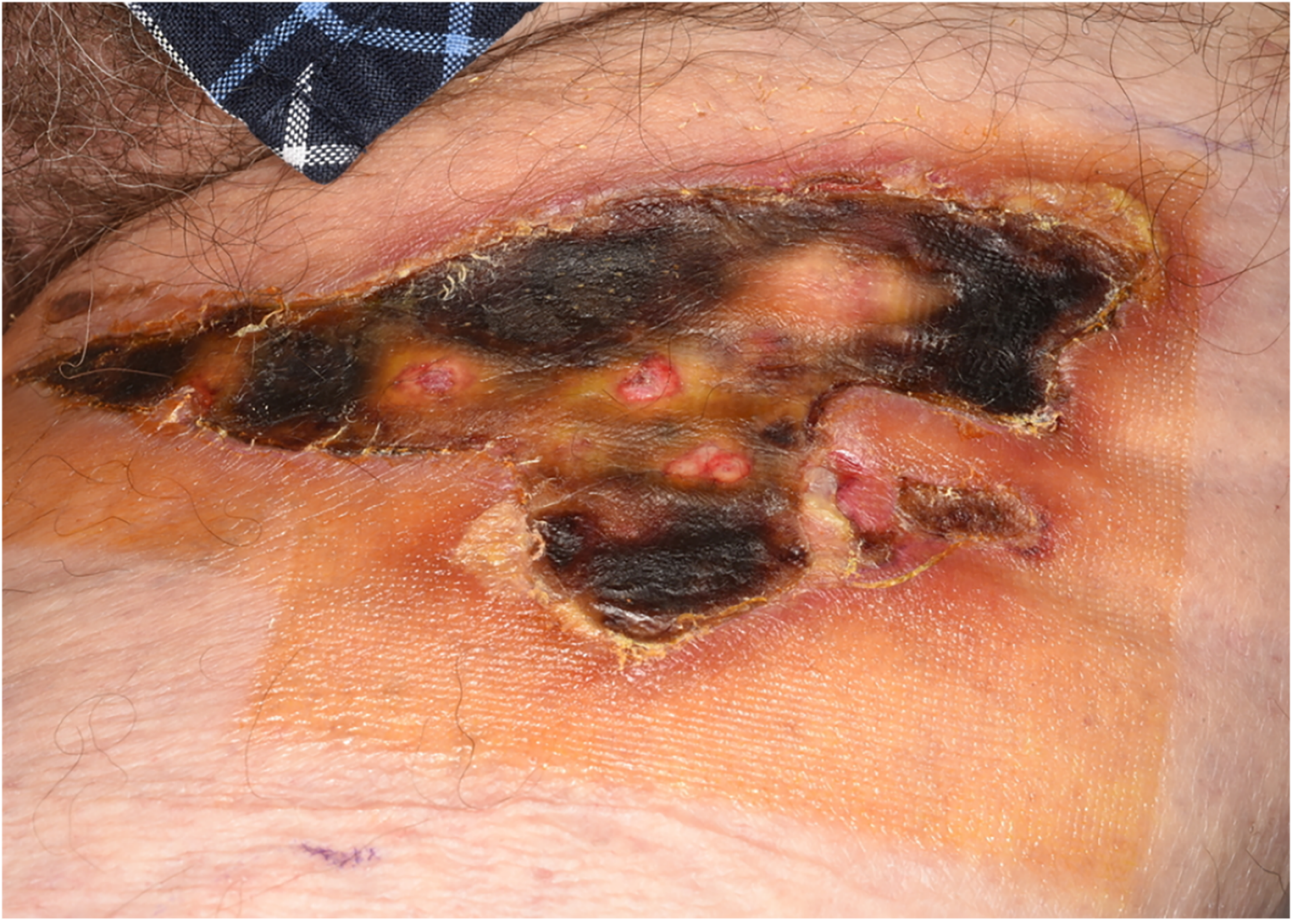
Day 14.

**Figure 3.**
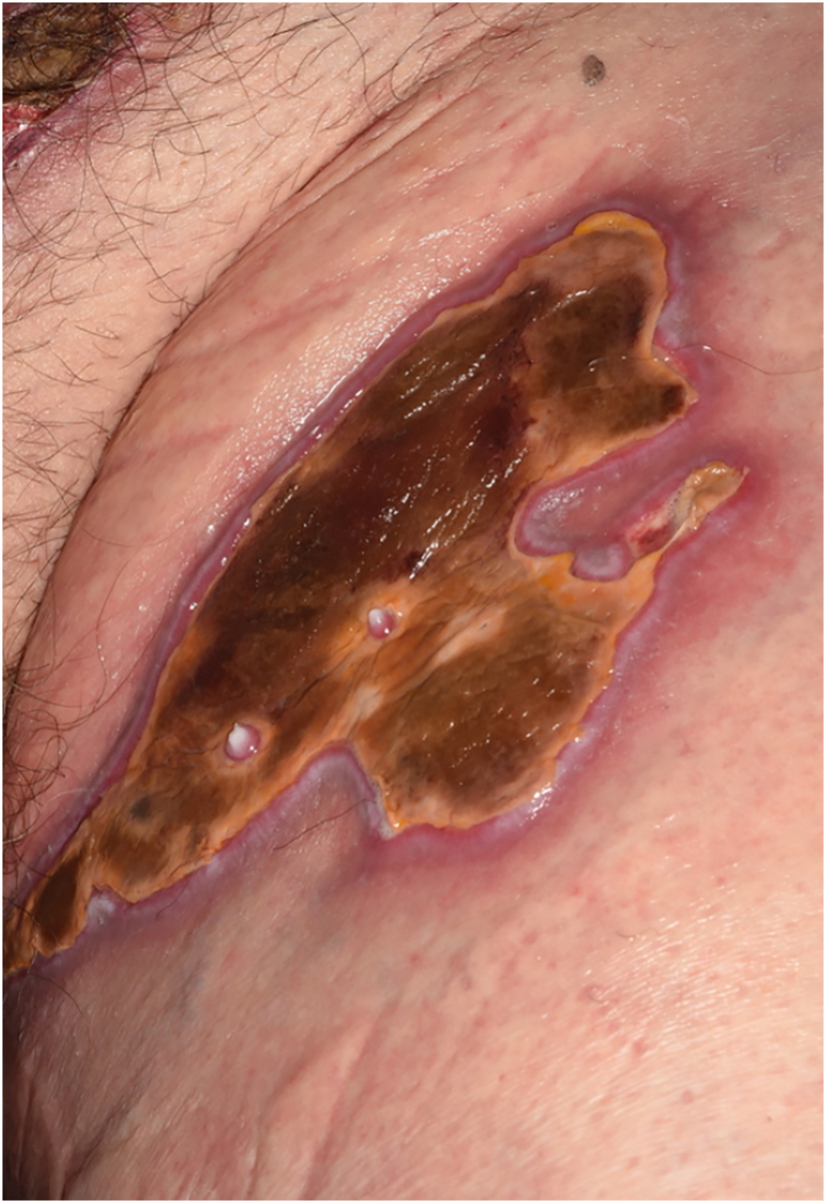
Day 21.

**Figure 4.**
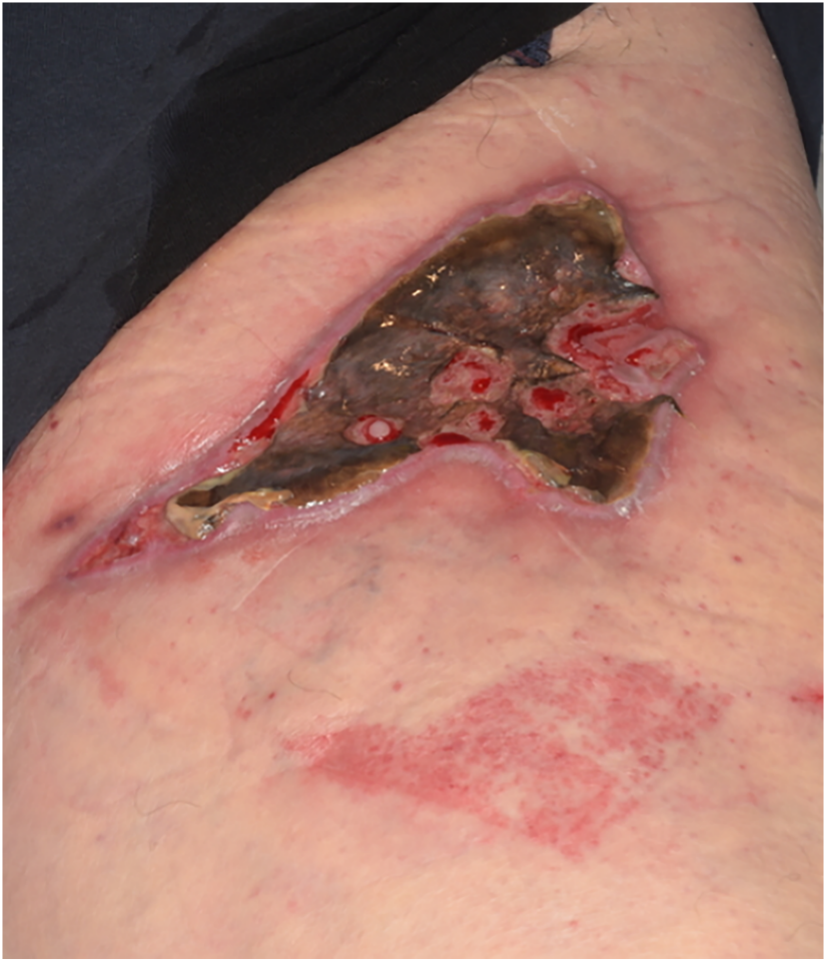
Day 33.

**Figure 5.**
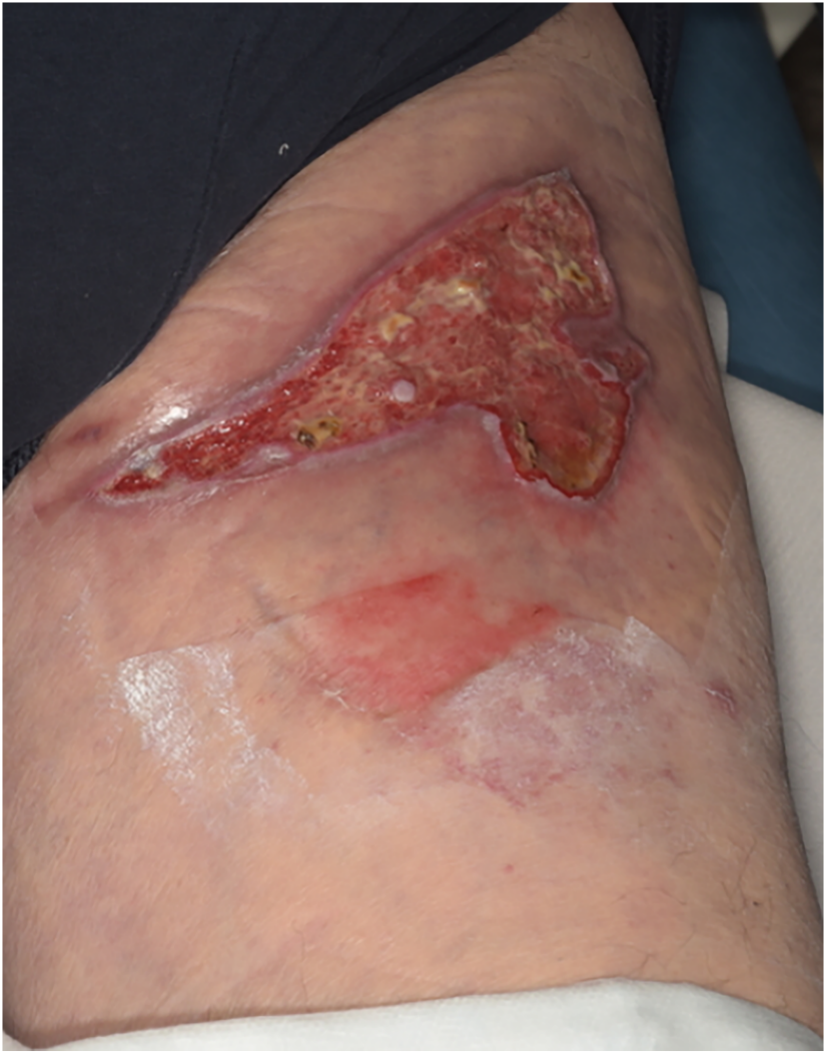
Day 37.

**Figure 6.**
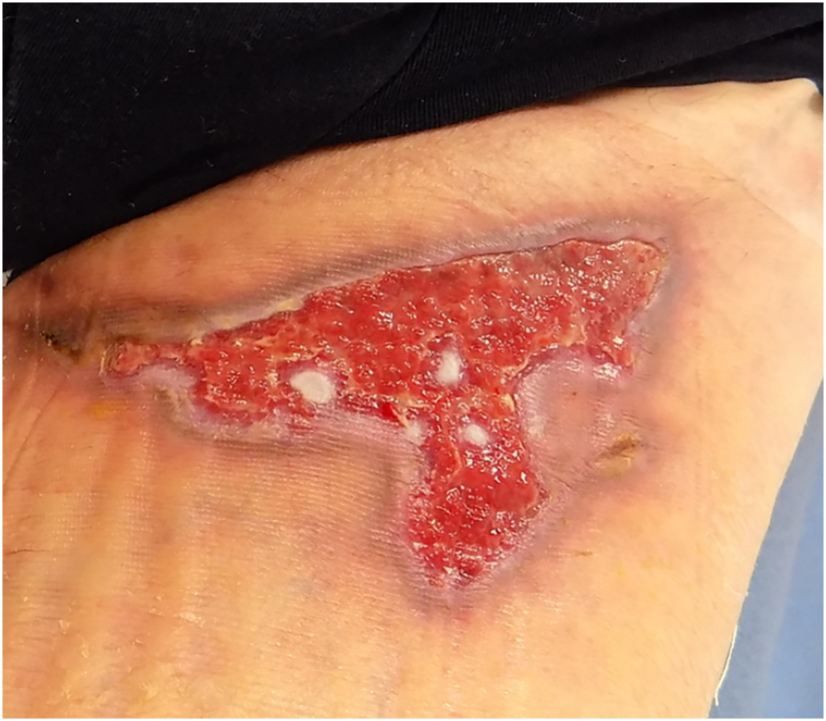
Day 51.

**Figure 7.**
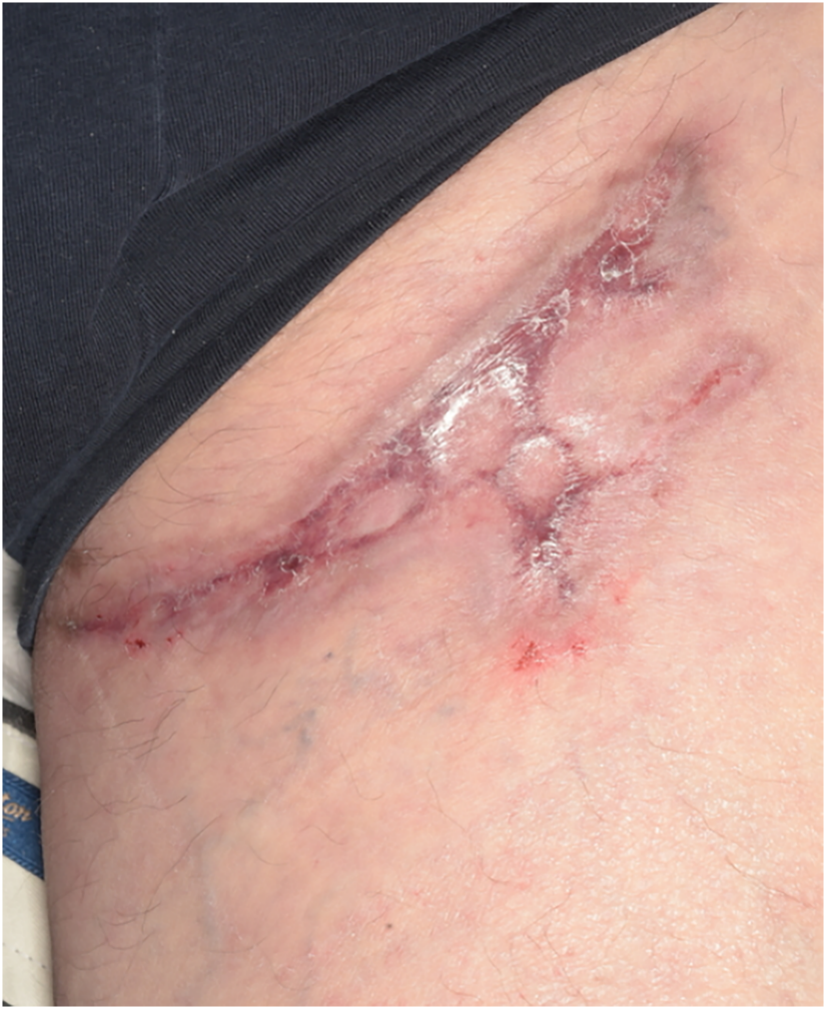
Day 86.

The patient was seen regularly in the outpatient burns clinic, and at 14 days his wound was deemed unlikely to heal and surgery was recommended. The patient did not want to have any kind of surgical procedure.

At three weeks, the decision was taken to try MDT, which was initiated at day 30 post-injury. He underwent a week of therapy with two rounds of MDT applied, before removal on day 37 and dressings applied. Within two months following MDT removal, his wound had healed completely and required regular dressing with simple non-woven dressings only.

## Patient perspective

***“*** *I had not realised how painful a mug of tea could be and the level of skill required to repair the damage… I was offered surgery with skin grafts or an option of sharing the wound with some lively wildlife. I chose the maggots for company as this seemed preferable. I was unaware of their presence until late some nights when they decided to let me know they were hungry. The maggots were extremely effective and achieved the results by speeding up the healing process quite dramatically”.*

## Discussion

We describe a case of MDT used successfully in the debridement of thick eschar in a full thickness burn wound as an alternative to surgical management. MDT resulted in effective, and relatively symptom free debridement. The patient was highly satisfied with the therapy as he was keen to avoid surgery.

The potential benefits of MDT in the treatment of burn wounds are manifold, but there is little evidence in the literature of its use for this purpose. MDT has been found to shorten time to healing, increase chances of healing and reduce antibiotics use in the treatment of ulcers, ischaemic wounds, traumatic wounds, and post-surgical wounds.^11^ Their use spans human history. Surgical debridement using sharp instruments has the disadvantage of debriding beyond the boundary of necrotic tissue so may not always be preferable.^17^ MDT minimises the trauma to healthy tissue.^11^ Maggots produce both a mechanical debridement using their mandibles and spines, but it is predominantly their secretion of proteolytic enzymes which break down eschar, induce fibrinolysis and therefore minimise inflammation and infection.^18^ These secretions may also degrade biofilms created by *Staphylococcus aureus* and *Pseudomonas aeruginosa*,^19^ which are the most ubiquitously cultured bacteria in burn wounds.^20^

The most common technique used for debridement of deep burn wounds with thick eschar is Bromelain-based enzymatic debridement (Nexobrid).^6^ However, it is recommended for use within four days of the initial injury. It should be used with caution in those taking anticoagulants,^20^ and its safety and efficacy has yet to be established in patients with diabetes. In our patient a decision was made for initial conservative therapy meaning that he fell out of the treatment window for Nexobrid.

With an increasingly ageing and comorbid population, MDT offers an alternative for the debridement of deep burn wounds with thick eschar, to allow preparation of the wound bed for surgical reconstruction and promote and expedite wound healing. MDT is associated with minimal symptoms and is well tolerated in the few cases reported in the literature. In this case, mild nocturnal discomfort only was reported on occasion, corroborating reports in the literature which describe MDT as a relatively pain free treatment modality, manageable with simple analgesia.^7,8,11,14^ In this case, two rounds of MDT resulted in successful eschar debridement, with larvae applied with a four-day interval and removed after three subsequent days. Two applications of MDT are commonly described as effective in achieving complete wound debridement,^16^ suggesting MDT offers a relatively quick and reportedly cost-effective method of debriding burns, even those complicated by hard, thick eschar. Although BioMonde BioBags are contraindicated in patients on anticoagulants, in this case the patient continued his anticoagulant during MDT without complications.

The few descriptions in the literature of the use of MDT in burns management focus on its role in debridement as a pre-grafting measure, to reduce perioperative risk and anaesthetic time. One report describes a series of elderly patients unsuitable for surgical intervention for whom MDT was successful.^14^ Wu et al. opted for MDT as an alternative to surgical debridement in a 60% burn to maximise the preservation of healthy tissue.^15^ A recent small randomised controlled trial compared the use of MDT versus conventional dressings with silver sulfadiazine prior to skin grafting.^21^ They observed hastened granulation in the MDT group, and importantly a reduced time to debridement and grafting and overall wound healing. However, this required intense wound care and three to four applications of larvae in patients who were planned for formal grafting anyway. We describe a case where MDT is used as a complete alternative to surgical management. This case highlights how MDT should be considered as a safe alternative to surgical debridement in the management of necrotic, full thickness burns with hard thick eschar, where MDT can effectively debride the wounds and facilitate and encourage effective wound healing, either as a pre-grafting measure or in place of a surgical approach.

## Barriers

MDT relies on prompt application following delivery and there can be a delay in sourcing the therapy. Each BioMonde Biobag costs between £258-£362 and must be ordered which can delay initiation.^22^ While in our case study, the patient was engaged with the therapy and even enjoyed the company, this attitude may not be shared by all patients. Due to limited evidence of their use, in burned tissue where the full depth of the wound is not explored there are risks associated with encountering superficial blood vessels, particularly if the patient is using anticoagulants. Evidence regarding the effect of antimicrobial compounds on maggot viability is limited,^23^ however manufacturers suggest compounds such as disinfectants, local anaesthetics, and hydrogels may negatively impact treatment.^24^

## Conclusion

MDT is a safe and effective means of both debriding and treating burn wounds and should be considered in the outpatient setting as an alternative to surgery. More evidence is required to compare healing rates, healing times and infection rates of MDT versus conventional therapies in burn wounds to inform clinicians and patients. This should ideally include the concomitant use of anticoagulants and antimicrobial dressings.
